# microRNA-21 promotes tumor proliferation and invasion in gastric cancer by targeting PTEN

**DOI:** 10.3892/or.2012.1645

**Published:** 2012-01-19

**Authors:** BAO GUI ZHANG, JIAN FANG LI, BEI QIN YU, ZHENG GANG ZHU, BING YA LIU, MIN YAN

**Affiliations:** Shanghai Key Laboratory of Gastric Neoplasms, Department of Surgery, Shanghai Institute of Digestive Surgery, Ruijin Hospital, Shanghai Jiao Tong University School of Medicine, Shanghai 200025, P.R. China

**Keywords:** gastric cancer, miR-21, biological behavior, PTEN

## Abstract

Gastric cancer is one of the most common carcinomas in China. microRNAs, a type of non-coding RNA, are important specific regulators and are involved in numerous bioprocesses of an organism. microRNA-21 (miR-21) has been identified as the most suitable choice for further investigation because it is overexpressed in nearly all solid tumors; furthermore, it has been demonstrated that miR-21 is involved in the genesis and progression of human cancer. It has been reported that PTEN, an important tumour suppressor, is regulated by multiple miRNAs. Thus, in this study we focused on the expression and significance of miR-21 in gastric cancer tissues, and the role of miR-21 in the biological behaviour and the expression of PTEN in gastric cancer cells. Real-time PCR was used to detect miR-21 expression in gastric cancer tissues, the adjacent normal tissues, and the gastric cell lines. The gastric cancer cell line BGC-823 was transfected with pre-miR-21/miR-21 inhibitor to overexpress/downregulate miR-21. The influence of miR-21 on the biological behaviour of gastric cancer cells was evaluated using the CCK-8 kit, FCMs, the scratch healing assay and the transwell test. Western blotting and the Luciferase Reporter Assay were used to evaluate the change of PTEN expression after lowered expression of miR-21 in gastric cancer cell lines. Real-time PCR analysis indicated that miR-21 exhibited higher expression in gastric cancer tissues compared to the adjacent non-tumor tissues. miR-21 expression was significantly associated with the degree of differentiation of the tumour tissues (P=0.004), as well as local invasion and lymph node metastasis (P<0.01). After transfection, pre-miR21 BGC-823 cells grew faster than the negative and control groups (P<0.01). The reduction in miR-21 expression demonstrated a remarkable effect on the biological behaviour of gastric cancer cells (P<0.05); the pre-miR-21-transfected cells healed more quickly compared to the control cells in the scratch healing assay, whereas the transwell test indicated that cell migration *in vitro* was notably inhibited with the downregulation of miR-21 (P<0.05). The western blot results and Luciferase Reporter Assay demonstrated that PTEN expression was remarkably increased after miR-21 inhibition (P<0.05). microRNA-21 expression was upregulated in gastric carcinoma tissues and was significantly associated with the degree of differentiation of tumour tissues, local invasion and lymph node metastasis. Overexpression of miR-21 promoted BGC-823 cell growth, invasion and cell migration *in vitro*, whereas downregulation of miR-21 exhibited a stronger inhibitory effect on the biological behaviour of gastric cancer cells; additionally, miR-21 inhibition may upregulate the PTEN expression level, which indicates that PTEN may be a target gene for gastric cancer initiation and development.

## Introduction

microRNA-21 negatively regulates several targets, and thus impacts tumorigenesis. However, the exact mechanism of action in human gastric carcinoma is poorly understood, and there is currently no direct evidence that demonstrates a correlation between microRNA-21 function and phenotype. In this study, we investigate the function of microRNA-21 as a potent oncomir and probe the relationship between microRNA-21, the targets of microRNA-21, and the phenotypic alterations. microRNAs (miRNAs) are small, non-coding RNAs of 18- to 23-nucleotides found in a diverse array of organisms. They have a broad impact on gene expression through translational repression or post-transcriptional suppression (1). microRNAs play an important role in numerous biological processes, such as development, differentiation, and the cellular stress response (2–4). Recent studies have linked deregulation of miRNAs to various diseases including cancer (5,6). Gastric cancer is the most common malignancy in China and the fourth most common cancer world-wide; moreover, the prognosis for gastric cancer is quite poor. Globally, it is the second and fourth leading cause of cancer-related death in men and women, respectively (7). Despite progress in the development of new management strategies, gastric cancer remains difficult to diagnose at an early stage. According to the International Union Against Cancer (UICC) and American Joint Committee on Cancer (AJCC) staging systems (8), nearly 65% of patients in the USA are initially diagnosed with gastric cancer at an advanced stage (T3/T4), and 85% of patients have lymph node metastasis (9). The mean survival time for these patients is 24 months and the 5-year survival rate is only 20–40% after surgery (10). Thus, the discovery of new biomarkers is of critical importance to allow for early diagnosis of gastric cancer.

Reports have indicated that dysregulation of miRNAs is associated with the formation and progression of gastric cancer (11,12). Therefore, miRNAs are potentially useful biomarkers for clinical diagnosis. Dramatic upregulation of microRNA-21 (miR-21) has been reported in numerous types of cancer (13–15). Therefore, miR-21 is recognized as an oncomir. Several targets of miR-21 have been experimentally validated, including PDCD4 (16) and RECK (17); however, ectopic expression of these targets may exert differing functional effects on tumorigenesis. Moreover, uncontrolled proliferation, lack of apoptosis, and invasiveness, which are all modulated by miR-21, have also been identified in tumorigenesis, leaving several questions unanswered. For example, PTEN is reported to be a direct target of miR-21 in HCC, but the phenotype alteration caused by miR-21-mediated PTEN regulation remains unclear. In this study, the mechanism of oncomir miR-21 in gastric cancer was studied with respect to the regulation of PTEN. We discovered that miR-21 expression was markedly increased in gastric cancer tissues compared to normal tissues. More importantly, we demonstrated that miR-21 promoted gastric cancer cell proliferation by directly targeting PTEN, thereby increasing gastric cancer cell invasiveness by directly targeting PTEN.

## Materials and methods

### Cell lines, cell culture, and human tissue samples

The human gastric cancer cell lines SGC-7901, MKN-28, MKN-45, and AGS were purchased from Shanghai Institutes for Biological Sciences, Chinese Academy of Sciences, and NCI-N87, BGC-823, HTB-103, CRL-5974, and CRL-5971 were purchased from the American Type Culture Collection (ATCC). The immortalized normal gastric mucosal epithelial cell line GES-1 was a gift from Professor Feng Bin (Sichuan University, Chengdu, China). The cells were routinely cultured in RPMI-1640 supplemented with 10% heat-inactivated fetal bovine serum (FBS), 100 U/ml penicillin and 100 μg/ml streptomycin in a humidified cell incubator with an atmosphere of 5% CO_2_ at 37°C. Cells growing at an exponential rate were used for the experiments.

Fresh frozen human tumor samples and human non-neoplastic gastric tissues were obtained from 30 patients with gastric cancer undergoing radical gastrectomy at the Department of Surgery, Ruijin Hospital Shanghai Jiao Tong University School of Medicine from May 2005 to August 2008. None of the patients had received preoperative treatment, such as radiation therapy or chemotherapy. Sections from each specimen were independently examined by two pathologists, and histological typing was performed using Lauren’s classification. TNM classification of malignant tumors was assigned in accordance to the International Union Against Cancer (1997).

### RNA isolation and miRNA cloning

Total RNA was extracted and isolated from tissue samples or cell lines using either the mirVana miRNA isolation kit (Ambion, Austin, TX) or the TRIzol method. The quality and quantity of the RNA samples were assessed by standard electrophoresis and spectrophotometric methods. The expression level of mature miR-21 was measured by qRT-PCR according to the TaqMan^®^ MicroRNA Assay protocol (Applied Biosystems) and normalized using U6 small nuclear RNA (RNU6B; Applied Biosystems) by the 2^−ΔCt^ method. The relative expression ratio of miR-21 in each paired tumour to non-tumour tissue sample was calculated using the 2^−ΔΔCt^ method. The miR-21 expression level was defined as being up-regulated in tumour tissue with a relative expression ratio >1, and was defined as downregulated in tumour tissue with a relative expression ratio <1.

### Transfection with antisense oligonucleotides

The stability-enhanced miRNA precursor that mimicks miR-21 and the control non-specific miRNA precursor (pre-miR precursor, negative control) and the miR-21 inhibitor (miR-21 inhibitor, negative control) were purchased from Shanghai GenePharma Co., Ltd. BGC-823 cells were trypsinised, counted, and seeded onto 6-well plates the day prior to transfection to ensure 50% cell confluence on the day of transfection. Transfection of miRNA precursors/inhibitors into BGC-823 cells was performed using Lipofectamine 2000 (Invitrogen) in accordance with the manufacturer’s advised procedure. The miRNA precursors/inhibitors were used at a final concentration of 100 nM. At 48 h post-transfection, qRT-PCR and western blot analysis were performed. Transfection efficiency was monitored by the transfection of Cy3-labeled pre-miR™ negative control #1 (Ambion).

### Cell proliferation assay

Cell proliferation was monitored by the colorimetric water-soluble tetrazolium salt (CCK8) assay using a Cell Counting Kit-8 (Dojindo) according to the manufacturer’s instructions. At 24 h post-transfection with miR-21 pre-cursor/inhibitor or control oligotides, BGC-823 cells were seeded onto 96-well plates (2×10^3^ cells/well), and cell proliferation was documented every 24 h for 4 days. The number of viable cells was assessed by measurement of the absorbance at 450 nm using a Safire^2^ microplate reader (TECAN).

### Cell cycle and apoptosis analysis

At 48 h post-transfection with the miR-21 precursor/inhibitor or control precursor (100 nM), BGC-823 cells were collected by trypsinisation and washed with phosphate-buffered saline (PBS). For cell cycle analysis, the cells were fixed with 75% ethanol and stored at 4°C overnight. The following day, fixed cells were washed with PBS, treated with RNase A (50 μg/ml), and stained with propidium iodide (PI) (50 μg/ml) for 30 min in the dark. The stained cells were analyzed by flow cytometry (FACSCalibur, Becton-Dickinson). The cell debris and fixation artifacts were gated out and the cell populations at the G0/G1, S, and G2/M phases were quantified using the Flowjo7.6.2 (Treestar). At least 10,000 cells in each sample were analyzed to obtain a measurable signal. For apoptosis analysis, an Annexin-V-FITC Apoptosis Detection Kit I (BD Pharmingen) was used according to the manufacturer’s instructions. In brief, cells were washed with PBS and resuspended in 1X binding buffer at a concentration of 1×10^6^ cells/ml; next, 5 μl of FITC Annexin-V and 5 μl PI were added to 100 μl of the cell suspension and the samples were incubated for 15 min in the dark, after incubation, 400 μl 1X binding buffer was added. Apoptosis was analyzed by flow cytometry (FACSCalibur, Becton-Dickinson) using the Cell-Quest software (Becton-Dickinson). The cells undergoing apoptosis were Annexin-V-FITC-positive and PI-negative.

### Cell migration assay

Migration of BGC-823 cells was assessed using the QCM™ 24-Well Colorimetric Cell Migration Assay Kit (Millipore) according to the manufacturer’s instructions. Briefly, at 24 h post-transfection with miR-21 inhibitor or control (100 nM), 2×10^4^ BGC-823 cells in 300 μl serum-free medium were added to the upper chamber. A volume of 0.5 ml of 10% FBS-containing medium was then added to the lower chamber as a chemoattractant. Cells were incubated for another 36 h at 37°C, and non-migrating cells on the upper surface of the membrane were then scraped off with cotton swabs. Cells that migrated to the bottom of the membrane were stained for 30 min with the cell stain provided in the assay kit. Stained cells were visualised under a microscope. To minimize the bias, at least three randomly selected fields were quantified using ×100 magnification, and the average number of cells was taken. For scratch wound-healing motility assays, BGC-823 cells were seeded on 6-well plates and allowed to grow to confluence. Confluent monolayers were scratched with a pipette tip and maintained under standard conditions for 24–48 h. Plates were washed once with fresh medium to remove non-adherent cells, and then photographed. Cells were treated with precursor (100 nM) for 24 h before wounding as well as throughout the assay period.

### Cloning of 3′UTR of PTEN into pMIR-REPORT luciferase vector

Total-RNA from BGC-823 cells was initially reverse transcribed into cDNA with Oligo(dT)_16_, which was used as the template, and wild-type and mutant PTEN 3′-UTRs were amplified by 5′-GGCACTAGTTATACTGGT TCACATCCTACCCCTTTGCACTTGTGGCAACAGAT AAGTTTGCAGTTGGCTAAGAGAGGTT-3′, and 5′-GAT AAGCTTCATTCCCCTAACCCGAATACATGCATTAG AATGTAGCAAAACCCTTCGGAAACCTCTCTTAGCC AACTGC-3′; 5′-GGCACTAGTTATACTGGTTCAC ATCCTACCCCTTTGCACTTGTGGCAACAGCTGAAT CTGCAGTTGGCTAAGAGAGGTT-3′, and 5′-GATAAG CTTCATTCCCCTAACCCGAATACATGCATTAGAAT GTAGCAAAACCCTTCGGAAACCTCTCTTAGCCAAC TGC-3′. The final pieces of wild-type and mutant PTEN 3′-UTRs were cloned into the *Spe*I and *Hin*dIII sites of the pMIR-REPORT luciferase vector (Ambion) and named pMIR/PTEN and pMIR/PTEN/mut, respectively. Both constructs were verified by sequencing.

### Luciferase activity assay

BGC-823 cells were cultured in 6-well plates, and each was transfected with 1 μg of either pMIR/PTEN vector or pMIR/PTEN/mut vector containing Firefly luciferase along with 0.05 μg of the pRL-TK vector (Promega) containing Renilla luciferase and 30 nM miR-21 inhibitor or control oligonucleotide. Transfection was performed using Lipofectamine 2000 (Invitrogen). At 24 h post-transfection, relative luciferase activity was calculated by normalising the Firefly luminescence to the Renilla luminescence using the Dual-Luciferase Reporter Assay (Promega) according to the manufacturer’s instructions.

### Western blot analysis and immunohistochemistry

Cultured cells were lysed using RIPA buffer (Pierce) in the presence of Protease Inhibitor Cocktail (Pierce). Tissue samples were lysed using the T-PER Tissue Protein Extraction Reagent (Pierce) in the presence of Protease Inhibitor Cocktail (Pierce). The protein concentration of the lysates was measured using a BCA Protein Assay Kit (Pierce). Equivalent amounts of protein were resolved and mixed with 5X Lane Marker Reducing Sample Buffer (Pierce), electrophoresed in a 12.5% SDS-acrylamide gel, and transferred to Immobilon-P Transfer Membrane (Millipore). The membranes were blocked with 5% non-fat milk in Tris-buffered saline and then incubated with mouse anti-human PTEN monoclonal antibody (Abcam) followed by horseradish peroxidase-conjugated secondary antibody (Abcam). Signals were detected with Immobilon Western chemiluminescent HRP Substrate (Millipore). GAPDH (Abcam) served as the loading control.

Tissues were fixed in 10% neutralised formalin and embedded in paraffin blocks. Sections (4 μm) were then prepared for immunohistochemical examination. After deparaffinisation and rehydration, antigen retrieval was performed by boiling in 10 mmol/l of citrate buffer (pH 6.0) for 10 min. After inhibition of endogenous peroxidase activity for 30 min with methanol containing 0.3% H_2_O_2_, the sections were blocked with 2% bovine serum albumin in PBS for 30 min and incubated with mouse anti-human PTEN monoclonal antibody (Abcam, dilution 1:500). The immune complex was visualised with the Dako REAL™EnVision™ Detection System, Perox-idase/DAB, Rabbit/Mouse (Dako), according to the manufacturer’s procedure. The cytoplasm was counterstained with hematoxylin.

### Statistical analysis

The relationships between the miR-21 expression level and clinicopathological parameters were analysed using the Pearson χ^2^ test. For comparisons between two different groups, statistical significance was determined using the Student’s t-test. All statistical analyses were performed using the SAS 6.12 software package. A two-tailed value of P<0.05 was considered statistically significant.

## Results

### The expression of miR-21 is upregulated in gastric cancer and correlates with clinicopathological parameters

**Figure 1:**
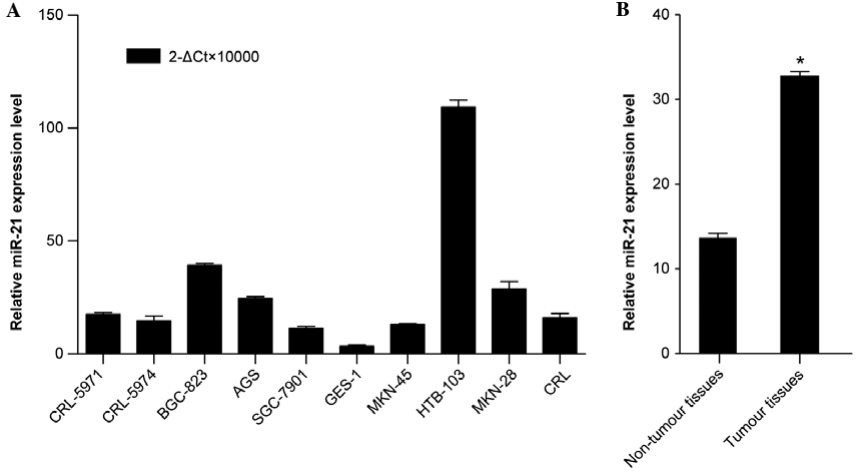
Upregulation of miR-21 expression in gastric cancer tissues and gastric cancer cell lines compared with the corresponding controls. (A) qRT-PCR for miR-21 was performed using nine gastric cancer cell lines and one immortalised normal gastric mucosal epithelial cell line (GES-1). The mean and standard deviation of miR-21 expression levels relative to the miR-21 expression level of GES-1 are shown. The data represent triplicate measurements from single RNA samples (P<0.05, compared with GES-1). (B) qRT-PCR for miR-21 was performed using 30 surgical specimens of gastric cancer tissues and matched with non-tumour tissues. The mean and standard deviation of miR-21 expression levels are shown. The data represent triplicate measurements from single RNA samples (P<0.05).

To explore the role of miRNAs in gastric cancer, the expression level of miRNAs was a primary consideration. We used miRNA microarray to analyse the miRNA expression profile of nine gastric cancer cell lines and six normal gastric mucosa, and we identified 146 under-expressed and 17 over-expressed miRNAs in all of the gastric cancer cell lines. It has been previously reported that some dysregulated miRNAs, such as miR-106a, miR-141, miR-143, miR-145, miR-218, miR-31, Let-7a, miR-17-5p, miR-221, miR-93, and miR-136 are altered in gastric cancer (18–24), which is in concordance with our results. Interestingly, we found significant upregulation of miR-21 in gastric cancer cell lines, which has not been previously described. Furthermore, qRT-PCR was performed to detect the miR-21 expression level in the nine gastric cancer cell lines in order to validate the high-expression trend of miR-21 in the gastric cancer cell lines obtained from the miRNA microarray analysis; the results were then compared to one immortalized normal gastric mucosal epithelial cell line (GES-1). As shown in Fig. 1A, miR-21 was significantly upregulated in most gastric cancer cell lines compared with GES-1, thus validating the miRNA microarray results. Similarly, by analyzing the clinical gastric cancer tissues, we found that the average expression level of miR-21 was also significantly upregulated in tumour tissues compared with its matched non-tumour tissues (Fig. 1B). Collectively, these results provided strong evidence that miR-21 is prominently upregulated in gastric cancer. Extensive analysis indicated that among the 30 gastric cancer tissues, 80% (24/30) of the tumour tissues exhibited upregulation of miR-21 compared with the matched non-tumour samples (relative expression ratio >1.0). Furthermore, 55% (16/30) of the tumour tissues exhibited greater significant upregulation of miR-21 (relative expression ratio >2.0). Based upon a relative expression ratio of >2.0, the miR-21 high-expression group demonstrated a trend toward differentiation. However, miR-21 expression demonstrated no relationship with age, gender, tumour site, or TNM stage (Table I).

### Ectopic expression of miR-21 promotes/inhibits the growth of gastric cancer cells

**Figure 2:**
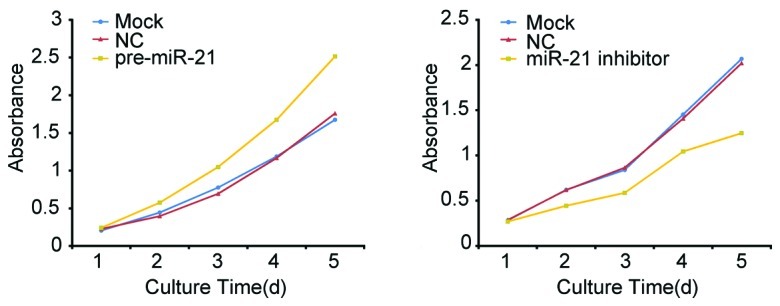
The effect of miR-21 on the proliferation of BGC-823 cells. Cell proliferation was measured by the CCK8 assay. BGC-823 cells were transfected with miR-21 precursor/inhibitor or control at a final concentration of 100 nM and, at 24 h post-transfection, the CCK8 assay was performed every 24 h for 4 days. Results are the mean of three independent experiments ± SD (P<0.05). The Cy3-labeled pre-miR™ negative control #1 was transfected into BGC-823 cells, and the transfection efficiency was assessed by fluorescence microscope (nuclei were stained with DAPI). Nearly all cells exhibited Cy3 staining, indicating that the miR-21 precursor/inhibitor and control were readily transfected into BGC-823 cells.

Because miR-21 is markedly upregulated in gastric cancer, it may thus function as a tumour promoter. Therefore, we tested whether over-expression/low-expression of miR-21 in BGC-823 cells affects cell growth. In a CCK8 assay, cells transfected with miR-21 precursor/inhibitor grew more rapidly/slowly than the control group (Fig. 2). The dramatic contrast in proliferative activity indicates that over-expression/low-expression of miR-21 promotes/inhibits the gastric cancer BGC-823 cell growth activity. These results suggest that over-expression/low-expression of miR-21 promotes/inhibits cell growth i*n vitro*. The transfection efficiency was monitored using a Cy3-labeled pre-miR™ negative control.

### Low-expression of miR-21 induces cell cycle arrest in G1/S phase and affects gastric cancer cell apoptosis

**Figure 3:**
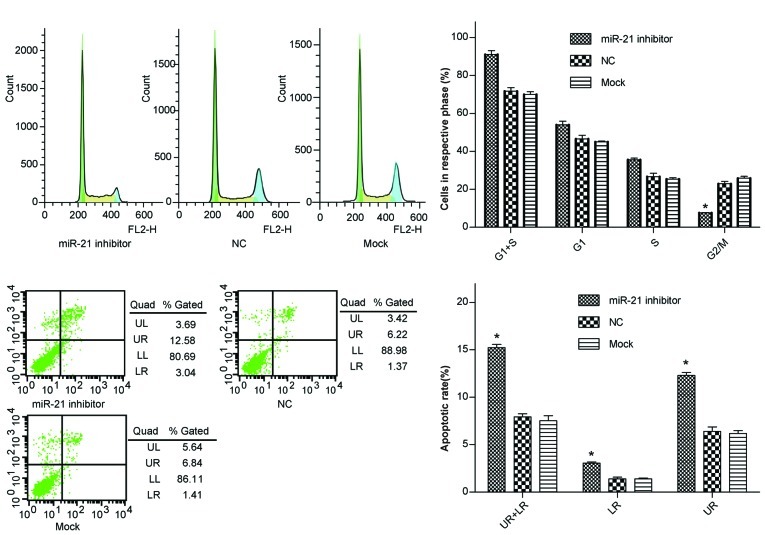
The effect of miR-21 on cell cycle distribution and apoptosis of BGC-823 cells. (A) Proportion of cells in various phases of the cell cycle. (B) Representative histograms depicting cell cycle profiles of BGC-823 cells transiently transfected with miR-21 inhibitor or control (100 nM). Cells were stained with PI and analysed by flow cytometry at 48 h post-transfection. The results are the mean of three independent experiments ± SD (P<0.05). (C) Cells staining positive for Annexin-V-FITC and negative for PI at 48 h post-transfection were considered to have undergone apoptosis. (D) Representative histograms depicting apoptosis of BGC-823 cells transiently transfected with miR-21 inhibitor or control (100 nM). Average apoptotic rate of three independent experiments ± SD are shown.

To elucidate the mechanism of miR-21-mediated cell growth promotion in gastric cancer cells, cell cycle analysis was performed. The results demonstrated that, when compared with the control group, the percentage of miR-21 inhibitor-transfected BGC-823 cells in G1/S phase increased from 70% to 91% (P<0.05), whereas the percentage of cells in G2/M phase decreased from 22.9 to 7.6% (P<0.05) (Fig. 3A and B), and there was a significant difference in the apoptotic rate between the differently treated groups (12.3 vs. 6.4%, P<0.05) (Fig. 3C and D). These results indicate that low-expression of miR-21 induces G1/S phase arrest in BGC-823 cells, which in turn contributes to the stimulating growth properties of miR-21.

### Ectopic expression of miR-21 promotes/inhibits migration of gastric cancer cells in vitro

**Figure 4:**
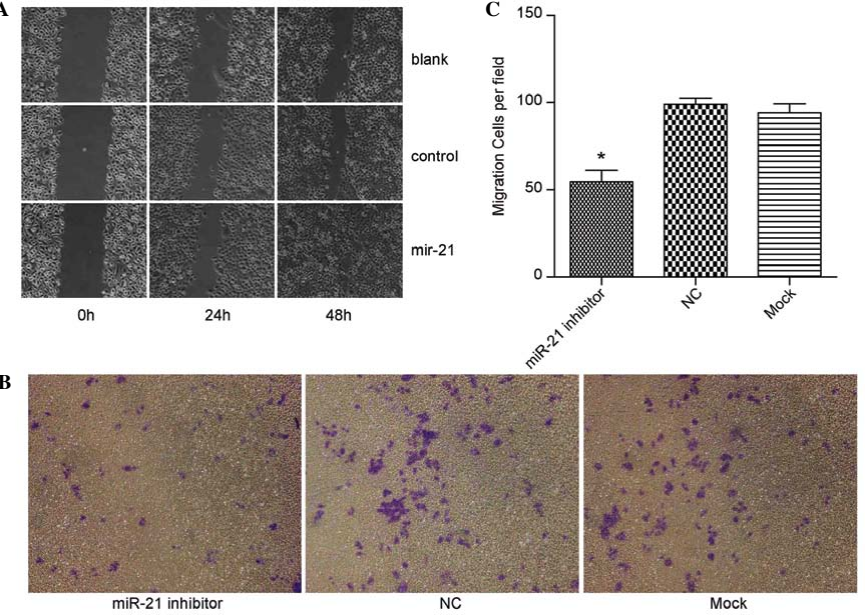
microRNA-21 promotes migration of BGC-823 cells *in vitro*. BGC-823 cells were first transfected with miR-21 precursor/inhibitor and control (100 nM) and then subjected to scratch wound-healing motility assays/transwell migration assays. (A) Representative photographs of Scratch wound-healing motility assays. (B and C) Average migratory cell number of three independent experiments ± SD (P<0.05).

We further assessed the effects of miR-21 on cell migration, a key determinant of malignant progression and metastasis. As shown in Fig. 4A, miR-21 precursor group scratch wound-healing motility was faster compared to the control group; (Fig. 4B and C) ectopic expression of miR-21 led to significantly decreased migration (miR-21 inhibitor group, 50±18 cells per field; control inhibitor group, 100±28 cells per field, P<0.05) of BGC-823 cells. These results propose a functional role for miR-21 in mediating cell migration in gastric cancer and suggest a mechanism by which upregulation of miR-21 potentially contributes to tumour metastasis in gastric cancer.

### PTEN is a target of miR-21

**Figure 5:**
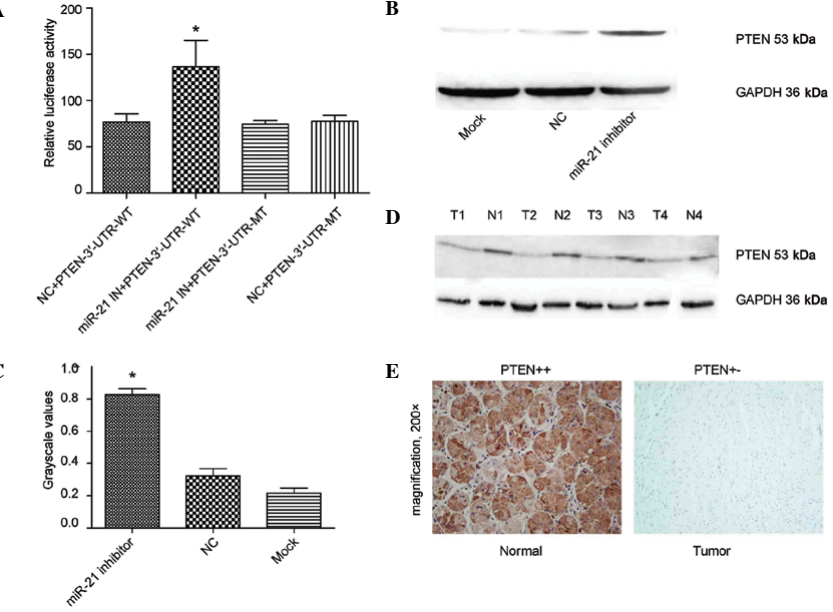
PTEN is a validated target of miR-21. (A) miR-21 inbibitor upregulated luciferase activities controlled by wild-type PTEN-3′-UTR (P<0.05) but did not affect luciferase activity controlled by mutant PTEN-3′-UTR. The results are the mean of three independent experiments ± SD (P<0.05). (B and C) PTEN protein in BGC-823 cells was detected by western blot analysis at 48 h post-transfection with miR-21 inhibitor and control (100 nM). GAPDH was used as an internal loading control. A reproducible result was obtained in three independent experiments. The results are shown as fold-changes relative to the control inhibitor-transfected BGC-823 cells. (D and E) PTEN expression in gastric cancer tissues and non-tumour tissues determined by western blot analysis and immunohistochemistry.

Because miR-21 has a pivotal function in gastric cancer, we investigated how this miRNA exerts its role in gastric cancer. We searched for further information regarding its potential target genes that exhibit anti-oncogene properties (13–15); among these genes, PTEN plays a crucial role in the signaling pathways regulating cell adhesion, proliferation, and migration. Therefore, we were able to confirm whether PTEN was also the authentic target gene of miR-21 in gastric cancer. To experimentally validate whether PTEN was a target gene of miR-21 in gastric cancer, a region of the PTEN-3′-UTR mRNA was cloned down stream of the Firefly luciferase gene in the pMIR-REPORT luciferase vector. This reporter construct (pMIR/PTEN) was co-transfected into BGC-823 cells with pRL-TK (containing the Renilla luciferase gene to normalize for transfection differences) and miR-21 inhibitor or control. A statistically significant increase of Firefly luciferase activity was observed in BGC-823 cells co-transfected with miR-21 inhibitor and pMIR/PTEN compared with BGC-823 cells co-transfected with control and pMIR/PTEN (Fig. 5A). The resulting construct, pMIR/PTEN/mut, was co-transfected into BGC-823 cells with miR-21 inhibitor or control, and the luciferase activity was measured. Importantly, miR-21 could no longer increase Firefly luciferase activity of pMIR/PTEN/mut (Fig. 5A). These results were confirmed by the following western blot analysis and immunohistochemistry (Fig. 5B-E). Taken together, these results indicate that PTEN-3′-UTR carries the direct binding sites of miR-21.

## Discussion

One of the best ways to understand miRNA function is the elucidation of functional targets, and this usually involves analysis of changes in target proteins following either a gain or loss of function of the specific miRNA. Our results indicate that miR-21 may regulate cell proliferation, apoptosis, and invasiveness by targeting PTEN. These results are somewhat similar to the findings in other types of cancer. However, there is no direct evidence to support a complex correlation among miRNAs, altered cell phenotype resulting from ectopic miRNA treatments, and the many targets of the miRNAs. We found an increase expression of a target gene (PTEN) that resulted from miR-21 inhibition; additionally, we found the inhibition of gastric cancer cell proliferation and the acceleration of apoptosis via anti-miR-21. Thus, we demonstrated that miR-21 generally handles different gastric cancer cell phenotypes by affecting the target, and that one target may contribute to several gastric cancer phenotypes under the control of miR-21. PTEN is a well-known tumor suppressor in multiple cancers, including HCC, and it affects the Akt and ERK signaling pathways (25–27). These pathways are linked to cell survival, proliferation, differentiation, cell migration, and invasion. Hence, this tumor suppressor could either be directly regulated by miR-21 or indirectly through the effect of miR-21 on PTEN. Although PTEN as a target gene of miR-21 has been validated in hepatocellular cancer, breast cancer, and non-small cell lung cancer (5,28,29), Hatley *et al* (5) confirmed that PTEN was not regulated by niR-21 in non-small cell lung cancer. Additionally, Medina *et al* (30) demonstrated that through over-expression, miR-21 could lead to pre-B-cell lymphoma, however, Hatley *et al* (5) suggested that miR-21 high-expression was not sufficient in the non-small cell lung cancer tumorigenesis model. Thus, how do we determine whether miR-21 is tissue specific? We observed an increase of PTEN expression in gastric cancer cells treated with anti-miR-21 as compared to untreated cells or cells treated with the scrambled-sequence oligonucleotide. These findings further demonstrate that miR-21, along with its known targets and a few associated genes, forms a complex regulatory network that plays an important role in gastric cancer formation and progression. Therefore, we believe that miR-21 is at the core of a tumorigenic regulatory network at a non-coding RNA level, and may be a new potential target for gastric cancer therapy. Taken together, our findings suggest that the phenotypes of gastric cancer cells (uncontrolled proliferation, increased survival, and invasiveness) are at least partly the result of miR-21 regulation of PTEN. Consequently, suppression of miR-21 may be a novel approach for the treatment of gastric cancer.

## Figures and Tables

**Table I:** Relationship between miR-21 expression level and clinicopathological parameters.

Clinicopathological parameters	n	mean ± SD	P-value
Age (years)			
≤59	14	9.00±9.81	0.675
>59	16	7.60±8.27	
Gender			
Male	17	10.±10.36	0.104
Female	13	5.42±5.71	
Bormann type			
I, II	6	7.23±8.03	0.758
III, IV	24	8.51±9.23	
Location			
Middle proximal	10	9.25±9.76	0.672
Distal	20	7.75±8.64	
Diameter (cm)			
≤5	18	10.58±10.24	0.138
>5	12	5.53±5.67	
Histological type			
Intestinal	10	9.17±9.25	0.562
Diffuse	20	7.54±8.32	
Depth of invasion			
T1, T2	8	2.23±4.16	0.042
T3, T4	22	9.8±10.36	
Lymph node metastasis			
No	9	3.13±2.17	0.028
Yes	21	10.3±9.67	
Differentiation			
High/middle	9	3.24±1.47	0.004
Moderate/low	21	10.4±9.88	
TNM stage			
I, II	7	5.22±7.76	0.312
III, IV	23	9.17±9.16	

The miR-21 expression level associated with clinicopathological features, including tumour size, lymph node metastasis, local invasion, and tumour-node-metastasis (TNM) stage are shown. Statistical significance was assessed by Pearson χ^2^ test.
